# Dr. Bibb and the Mexican National Railroad

**Published:** 1897-08

**Authors:** 


Dr. Bibb and the Mexican National Railroad.—During a recent visit to the City of Mexico, the writer had the pleasure of meeting his old friend, the *Journal's* correspondent, Dr. R. H. L. Bibb, formerly of Texas, and a long time secretary of the Texas State Medical Association. Dr. Bibb is chief surgeon of the Mexican National railroad, the most direct route to the City of Mexico, and 256 miles shorter route than any other. It is a narrow guage road, and is a perfect little gem; handsomely and thoroughly equipped with every convenience and comfort for traveling, it is a positive luxury to go over it. The roadbed is solid and level, and the trains move with the smoothness and velocity of a bicycle. No accident has ever happened to a passenger on this road in the seventeen years it has been in operation. The road takes one through the most beautiful and picturesque portions of the Republic and through most of the historic places.

Dr. Bibb is also surgeon of the American hospital in the City of Mexico. He is also doing a splendid practice. His many friends will be pleased to hear of his prosperity. We regret to state that recently the doctor has experienced some impairment of his general health—some digestive trouble—and has lost about thirty pounds of flesh. But the loss is "becoming" to his personal appearance; he is even finer looking than ever. During our visit the doctor placed us under additional obligations by kindly and courteous attentions, which we shall not forget.

